# Mcl‐1 and Bcl‐xL levels predict responsiveness to dual MEK/Bcl‐2 inhibition in B‐cell malignancies

**DOI:** 10.1002/1878-0261.13153

**Published:** 2021-12-18

**Authors:** Katrine Melvold, Mariaserena Giliberto, Linda Karlsen, Pilar Ayuda‐Durán, Robert Hanes, Toril Holien, Jorrit Enserink, Jennifer R. Brown, Geir E. Tjønnfjord, Kjetil Taskén, Sigrid S. Skånland

**Affiliations:** ^1^ Department of Cancer Immunology Institute for Cancer Research Oslo University Hospital Norway; ^2^ K. G. Jebsen Centre for B Cell Malignancies Institute of Clinical Medicine University of Oslo Norway; ^3^ Institute of Clinical Medicine University of Oslo Norway; ^4^ Faculty of Medicine Centre for Cancer Cell Reprogramming Institute of Clinical Medicine University of Oslo Norway; ^5^ Department of Molecular Cell Biology Institute for Cancer Research Oslo University Hospital Norway; ^6^ Department of Clinical and Molecular Medicine NTNU – Norwegian University of Science and Technology Trondheim Norway; ^7^ Department of Immunology and Transfusion Medicine St. Olav’s University Hospital Trondheim Norway; ^8^ Department of Hematology St. Olav’s University Hospital Trondheim Norway; ^9^ Faculty of Mathematics and Natural Sciences Department of Biosciences University of Oslo Norway; ^10^ Department of Medical Oncology Dana‐Farber Cancer Institute Boston MA USA; ^11^ Harvard Medical School Boston MA USA; ^12^ Department of Haematology Oslo University Hospital Norway

**Keywords:** cell signaling, chronic lymphocytic leukemia, drug sensitivity, mantle cell lymphoma, MEK inhibitors, multiple myeloma, phospho flow, synergy, venetoclax

## Abstract

Most patients with chronic lymphocytic leukemia (CLL) initially respond to targeted therapies, but eventually relapse and develop resistance. Novel treatment strategies are therefore needed to improve patient outcomes. Here, we performed direct drug testing on primary CLL cells and identified synergy between eight different mitogen‐activated protein kinase kinase (MEK) inhibitors and the B‐cell lymphoma 2 (Bcl‐2) antagonist venetoclax. Drug sensitivity was independent of immunoglobulin heavy‐chain gene variable region (IGVH) and tumor protein p53 (TP53) mutational status, and CLL cells from idelalisib‐resistant patients remained sensitive to the treatment. This suggests that combined MEK/Bcl‐2 inhibition may be an option for high‐risk CLL. To test whether sensitivity could be detected in other B‐cell malignancies, we performed drug testing on cell line models of CLL (*n* = 4), multiple myeloma (MM; *n* = 8), and mantle cell lymphoma (MCL; *n* = 7). Like CLL, MM cells were sensitive to the MEK inhibitor trametinib, and synergy was observed with venetoclax. In contrast, MCL cells were unresponsive to MEK inhibition. To investigate the underlying mechanisms of the disease‐specific drug sensitivities, we performed flow cytometry‐based high‐throughput profiling of 31 signaling proteins and regulators of apoptosis in the 19 cell lines. We found that high expression of the antiapoptotic proteins myeloid cell leukemia‐1 (Mcl‐1) or B‐cell lymphoma‐extra large (Bcl‐xL) predicted low sensitivity to trametinib + venetoclax. The low sensitivity could be overcome by combined treatment with an Mcl‐1 or Bcl‐xL inhibitor. Our findings suggest that MEK/Bcl‐2 inhibition has therapeutic potential in leukemia and myeloma, and demonstrate that protein expression levels can serve as predictive biomarkers for treatment sensitivities.

## Graphical abstract

Combined treatment with a MEK inhibitor and the Bcl‐2 antagonist venetoclax was effective in chronic lymphocytic leukemia cells, independently of high‐risk prognostic markers. The combination was effective also in multiple myeloma, but not in mantle cell lymphoma cells. High expression of Mcl‐1 or Bcl‐xL predicted the low sensitivity, which could be overcome by adding in an Mcl‐1 or Bcl‐xL inhibitor.

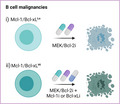

**Abbreviations**
AUC: area under the curve
Bcl‐2: B‐cell lymphoma 2
Bcl‐2i: Bcl‐2 inhibitor
Bcl‐xL: B‐cell lymphoma‐extra large
BTK: Bruton’s tyrosine kinase
CLL: chronic lymphocytic leukemia
DSS: drug sensitivity score
EMA: European Medicines Agency
ERK: extracellular signal‐regulated kinase
FDA: US Food and Drug Administration
IGVH: immunoglobulin heavy‐chain gene variable region
MAPK: mitogen‐activated protein kinase
MCL: mantle cell lymphoma
Mcl‐1: myeloid cell leukemia‐1
MEK: mitogen‐activated protein kinase kinase
MEKi: MEK inhibitor
MM: multiple myeloma
n.s.: not significant
PBMCs: peripheral blood mononuclear cells
PI3K: phosphatidylinositol‐3 kinase
SEM: standard error of the mean
TP53: tumor protein p53

## 1 Introduction

The introduction of targeted therapies has greatly improved the management of hematological malignancies. Still, development of treatment resistance remains an unresolved challenge, stressing the need for novel drug targets and combinatorial approaches [1, 2, 3]. Direct drug testing of a patient’s tumor cells can identify effective therapies, including novel agents and combinations [4]. The feasibility of this approach has been demonstrated for aggressive hematological malignancies in the EXALT study (NCT03096821), in which integration of sensitivity testing in treatment decisions improved the treatment outcome [5]. The follow‐up study, EXALT‐2 (NCT04470947), is a randomized, three‐arm study, which compares treatment decisions guided by drug screening, genomic profiling, and physician’s choice. Results from this trial will guide future choice of approach to precision medicine.

The mitogen‐activated protein kinase (MAPK) signaling pathway controls cellular processes such as cell proliferation and programmed cell death [6]. The classic MAPK pathway consists of the proteins Ras, Raf, mitogen‐activated protein kinase kinase (MEK), and extracellular signal‐regulated kinase (ERK), which sequentially relay signals from the cell surface to the nucleus. The pathway is activated in numerous neoplasms, and MEK inhibitors (MEKi) have therefore been developed and studied as a therapeutic option in various clinical settings [7].

Trametinib was the first MEKi approved by the US Food and Drug Administration (FDA) and is the MEKi most commonly used in clinical studies (Table 1). Binimetinib and cobimetinib are also FDA‐approved MEKi and are, like trametinib, indicated for BRAF+ melanoma (Table 1). Several additional MEKi are in advanced stages of clinical testing (Table 1). While most studies are on solid tumors, a few ongoing trials study the MEKi binimetinib (NCT02089230), selumetinib (NCT00588809), and trametinib (NCT02016729, NCT00920140, NCT01428427, NCT01476137) in leukemia (Table 1). A recent study showed that chronic lymphocytic leukemia (CLL) patients resistant to the phosphatidylinositol‐3 kinase (PI3K) inhibitor idelalisib had activating mutations in MAPK pathway genes, providing a rationale for combined PI3K/MEK inhibition in this group of CLL patients [8]. Dual targeting of these pathways is currently studied in clinical trials on multiple myeloma (MM; NCT01476137, NCT01989598).

**Table 1:** MEKis included in the study^a^. EMA, European Medicines Agency; FDA, US Food and Drug Administration.

Drug	Clinical trials registered at clinicaltrials.gov	With venetoclax	Approved by (indication)
Binimetinib	102; 22 completed, 6 on leukemia (NCT04322383, NCT02089230, NCT02225574, NCT04324112, NCT02049801, NCT01885195)		EMA, FDA (BRAF+ melanoma)
Cobimetinib	107; 30 completed, 1 on leukemia (NCT02670044)	NCT03312530, NCT02670044	EMA, FDA (BRAF+ melanoma)
PD0325901	12; 1 completed		
Pimasertib	13; 8 completed		
Refametinib	8; 7 completed		
Selumetinib	120; 61 completed, 3 on leukemia (NCT03705507, NCT03326310, NCT00588809)		FDA (pediatric patients with neurofibromatosis type 1)
Trametinib	227; 63 completed, 9 on leukemia (NCT01907815, NCT03190915, NCT02016729, NCT04487106, NCT01428427, NCT00920140, NCT01376310, NCT03878524, NCT02551718)	NCT04487106	EMA, FDA (BRAF+ melanoma)
U0126			

^a^ As of June 2021.

Like other targeted therapies, MEKi are associated with characteristic adverse events [9]. If the MEKi can be administered as part of a combination regimen, synergy between the agents could justify a reduced drug dose, which may also lower toxicities. Preclinical studies have indicated synergy between MEKi and fludarabine, PI3K inhibitors, and B‐cell lymphoma 2 (Bcl‐2) antagonists in CLL [10, 11, 12].

Here, we set out to identify novel therapeutic options for CLL and related B‐cell malignancies. *Ex vivo* drug sensitivity screens on primary CLL cells revealed synergy between MEKi and venetoclax. Combined MEK/Bcl‐2 inhibition was effective in MM cell lines as well, but not in mantle cell lymphoma (MCL). Low basal phosphorylation levels of MAPK pathway proteins correlated with high efficacy of the MEKi trametinib, while high expression of the antiapoptotic protein myeloid cell leukemia‐1 (Mcl‐1) or B‐cell lymphoma‐extra large (Bcl‐xL) correlated with low sensitivity to MEK/Bcl‐2 inhibition. Interestingly, treatment with an Mcl‐1 or Bcl‐xL inhibitor sensitized the MCL cells toward MEK/Bcl‐2 inhibition.

Our findings suggest therapeutic activity of combined MEK/Bcl‐2 inhibitors in CLL and MM, and provide a mechanistic rationale for disease‐specific sensitivities to MEK/Bcl‐2 inhibition.

## 2 Materials and methods

### 2.1 Cell lines

The CLL cell lines used in this study were HG3, MEC1 (from R. Rosenquist, Karolinska Institutet, Sweden [13, 14]), OSU‐CLL (from J. Byrd, Ohio State University, USA [15]), and PCL12 (from C. Scielzo, The IRCCS San Raffaele Scientific Institute, Italy [16]). OSU‐CLL has mutated IGVH, while HG3, MEC1, and PCL12 have unmutated IGVH. The melanoma cell lines used in this study were A375, FEMX‐1, FEMX‐V, Lox, MeWo, WM9, WM239, WM266.4, WM983b, and WM1366 (from E. Hovig, Oslo University Hospital, Norway). The MM cell lines used were CAG [17], INA6 [18], JJN3 [19], KJON [20], OH‐2 [21], U‐266 [22], URVIN, and VOLIN [20]. The MCL cell lines GRANTA‐519, JEKO‐1, JVM‐2, MAVER‐1, MINO, REC‐1, and Z138 were purchased from DSMZ—German Collection of Microorganisms and Cell Cultures GmbH (Braunschweig, Germany), and generously provided by J. Myklebust, Oslo University Hospital, Norway. The cell lines were cultured in RPMI 1640 GlutaMAX medium (Thermo Fisher Scientific, Waltham, MA, USA) supplemented with 10% fetal bovine serum (FBS), 1% penicillin/streptomycin, 1× minimum essential medium nonessential amino acids (MEM NEAA), and 1× sodium pyruvate (NaP) (complete medium). The MM cell lines were cultured in complete medium supplemented with 2 mm l‐glutamine (Sigma‐Aldrich, Saint‐Louis, MO, USA) (all), and 15% FBS (U‐266 and URVIN) or 10% human serum (KJON, OH‐2, and VOLIN) instead of 10% FBS. The growth medium was supplemented with 2 ng·mL^−1^ interleukin‐6 (Thermo Fisher Scientific) for culturing of INA6, KJON, OH‐2, URVIN, and VOLIN. The cells were expanded, aliquoted, and cryopreserved until experimental assays were performed.

### 2.2 Patient material and ethical considerations

Buffy coats from age‐matched, anonymized healthy blood donors were obtained from the Department of Immunology and Transfusion Medicine at Oslo University Hospital. Blood samples from CLL patients were from the Department of Haematology, Oslo University Hospital, Norway, and Dana‐Farber Cancer Institute (DFCI), MA, USA. All donors included in the study gave a written informed consent prior to sample collection. The study was approved by the Regional Committee for Medical and Health Research Ethics of South‐East Norway (2016/947 and 28507). The DFCI tissue bank protocol was approved by the Dana‐Farber Harvard Cancer Center Institutional Review Board. Research on human blood was carried out in agreement with the Declaration of Helsinki.

### 2.3 Reagents and antibodies

Catalogue numbers are indicated in parentheses. A‐1331852 (S7801), binimetinib (S7007), cobimetinib (S8041), PD0325901 (S1036), pimasertib (S1475), refametinib (S1089), S63845 (S8383), selumetinib (S1008), U0126 (S1102), and venetoclax (S8048) were from Selleck Chemicals LLC (Houston, TX, USA). Trametinib (T‐8123) was from LC laboratories (Woburn, MA, USA). The complete drug library used in this study is depicted in Tables S1 and S2. The following Alexa Fluor 647‐conjugated antibodies were from Cell Signaling Technologies (Danvers, MA, USA): AKT (pT308) (3375), AKT (pS473) (4075), Bcl‐2 (82655), Bcl‐xL (86387), Bim (10408), cleaved caspase‐3 (Asp175) (clone D3E9) (9602), Mcl‐1 (78471), p38 MAPK (pT180/Y182) (4552), p44/42 MAPK (ERK1/2) (pT202/Y204) (4375), p90RSK (pS380) (13575), S6‐ribosomal protein kinase (pS235/S236) (4851), SAPK/JNK (pT183/Y185) (9257), SYK (pY525/Y526) (12081), and tyrosine (pY100) (9415). The following Alexa Fluor 647‐conjugated antibodies were from BD Biosciences (San Jose, CA, USA): Bcl‐2 (pS70) (562531), Bruton’s tyrosine kinase (BTK) (pY223)/ITK (pY180) (564846), BTK (pY551)/ITK (pY511) (558134), IgG1 K isotype control (557783), MEK1 (pS298) (560043), MEK1 (pS218)/MEK2 (pS222) (562460), mTOR (pS2448) (564242), NF‐κB p65 (pS529) (558422), p53 (pS37) (560280), PLCγ2 (pY759) (558498), Rb (pS807/S811) (558590), STAT1 (pY701) (612597), STAT1 (pS727) (560190), STAT3 (pY705) (557815), STAT3 (pS727) (558099), STAT5 (pY694) (612599), STAT6 (pY641) (612601), TBK1 (pS172) (558603), and ZAP70/SYK (pY319/Y352) (557817). PerCP‐Cy5.5‐conjugated mouse anti‐human CD19 (clone HIB19) was from eBioscience (San Diego, CA, USA). PE‐Cy7‐conjugated mouse anti‐human CD3 antibody (UCHT1) was from BD Biosciences. Goat F(ab')2 anti‐human IgM (used at 10 µg·mL^−1^) was from Southern Biotech (Birmingham, AL, USA). BD Phosflow Fix Buffer I and Perm Buffer III were from BD Biosciences. Fluorescent cell barcoding fluorochromes Alexa Fluor 488, Pacific Blue, and Pacific Orange succinimidyl ester were from Thermo Fisher Scientific.

### 2.4 Isolation of lymphocytes and peripheral blood mononuclear cells

Isolation of CD3^+^ T cells and CD19^+^ B cells from buffy coats from healthy blood donors, and peripheral blood mononuclear cells (PBMCs) from CLL patient samples, was performed as described [23, 24]. Isolated cells were cryopreserved in liquid nitrogen [24]. See Table 2 for patient characteristics.

**Table 2:** Patient characteristics. B, bendamustine; C, cyclophosphamide; F, fludarabine; R, rituximab; f, female; m. male; M, mutated IGVH; n.e., not established; OFA, ofatumumab; UM, unmutated IGVH.

Patient ID	Gender	Age	Binet stage	IGVH	FISH/TP53	Treatment at procurement	Treatment prior to procurement	Samples collected (if more than one)
CLL001D	M	68	A	M	n.e.			
CLL002D	M	66	A	UM	n.e.			
CLL003D	M	73	A	M	n.e.			
CLL150	F	64	C	UM	del(13q14), TP53 mutation	Venetoclax	FCR, FC, Ibrutinib, Idelalisib	
CLL152	F	56	B	UM	n.e.			
CLL153	M	51	C	UM	del(11q22), trisomy 12, del(13q14)			
CLL154	M	61	A	M	n.e.			
CLL159	M	66	A	M	n.e.			
CLL160	M	72	A	M	46,XY			
CLL161	M	55	A	M	n.e.			
CLL166	F	64	C	UM	del(6q23), TP53 mutation	Ibrutinib	F, BR, FCR, allotransplant, Ibrutinib	
CLL185	F	58	A	M	n.e.			
CLL216	M	58	C	M	del(13q14)		C, radiation, FCR	
JB‐0058	M	58, 61	A, C	UM	del(11q22), del(13q14)	Idelalisib	FCR	Responding, resistant
JB‐0157	F	71, 71, 73	C	M	del(13q14), del(17p), TP53 mutation	Idelalisib (with OFA at initiation)	BR	Baseline, responding, resistant
JB‐0158	M	53, 55, 57	C, A, A	M	Normal	Idelalisib (with OFA at initiation)	BR	Baseline, responding, resistant
JB‐0197	M	80, 82, 83	A, C, C	UM	trisomy 12, del(17p)	Idelalisib	BR	Baseline, responding, resistant
JB‐0237	M	66, 66	C, A	UM	del(13q14)	Idelalisib		Baseline, responding
JB‐0238	M	71, 72	C, A	M	del(13q14)	Idelalisib		Baseline, responding
JB‐0244	M	80, 80	C, A	UM	del(11q22), del(13q14), del(17p)	Idelalisib		Baseline, responding

### 2.5 CellTiter‐Glo luminescent cell viability assay

Experiments were performed as described previously [25]. Briefly, drugs (Table S1) were preprinted into 384‐well cell culture microplates with an acoustic liquid handling device (Echo 550, Labcyte Inc., San Jose, CA, USA). Each compound was tested at five different concentrations ranging from 1 nm to 10 000 nm. Combinations were designed using the fixed molar concentration series identical to those used for single agents. CLL cells were cocultured with CD40L+, BAFF+, and APRIL+ L cells (ratio 1 : 1 : 1) for 24 h prior to initiation of the experiment to mimic the tumor microenvironment and to prevent spontaneous apoptosis. The L cells were removed by positive selection using PE anti‐mouse CD47 antibody (Biolegend, San Diego, CA, USA) and anti‐PE microbeads (Miltenyi Biotec, Bergisch Gladbach, Germany) according to the manufacturer’s instructions. B and T cells from healthy donors were treated the same way. Single‐cell suspension (10 000 cells·well^−1^) was distributed to each well using the CERTUS Flex liquid dispenser (Fritz Gyger, Thun, Switzerland). Experiments on cell lines were performed on freshly thawed cells to reduce variation between experiments introduced by culturing conditions. The cells were incubated with drugs at 37 °C for 72 h. Cell viability was measured using the CellTiter‐Glo (Promega, Madison, WI, USA) luminescent assay according to the manufacturer’s instructions. Luminescence was recorded with an EnVision 2102 Multilabel Reader (PerkinElmer, Waltham, MA, USA). The response readout was normalized to the negative (DMSO) and positive (100 µm benzethonium chloride) controls. The raw dose–response data were processed with the KNIME software (KNIME AG, Zurich, Switzerland).

### 2.6 Phospho flow with fluorescent cell barcoding

Phospho flow experiments were performed on samples from CLL150, CLL153, and CLL216 (Table 2) and analyzed with a BD LSRFortessa cytometer (BD Biosciences) equipped with 488, 561, 640, and 407 nm lasers [26]. The data were analyzed in Cytobank (https://cellmass.cytobank.org/cytobank/) as described [27].

### 2.7 Data analysis and statistics

Output data from drug sensitivity screens and phospho flow experiments were processed in graphpad prism 8 (San Diego, CA, USA). Applied statistical tests and models are indicated in the figure legends. To quantitatively score the drug responses, a modified drug sensitivity score (DSS) was calculated for each sample and compound separately [28]. Area under the concentration–response curve (AUC) was calculated using an activity window from 100% to 10%, and a dose window from the minimum concentration tested to the concentration where the viability reached 10%. DSS_3_ metric was used, without the division by the logarithm of the upper limit of the logistic curve. The DECREASE tool was used to predict the full concentration–response matrices from normalized viability data [29]. These matrices were then provided as input to the SynergyFinder web tool (version 2.0; https://synergyfinder.fimm.fi) for analysis of drug synergy [30]. Bliss synergy was selected as output [31]. The net AUC values from phospho flow experiments were calculated in graphpad prism 8. ClustVis (https://biit.cs.ut.ee/clustvis/) was used for visualizing clustering of the phospho flow data [32]. Figure 5G was made with BioRender.com.

## 3 Results

### 3.1 MEKi synergize with venetoclax in primary CLL cells

**Fig. 1:**
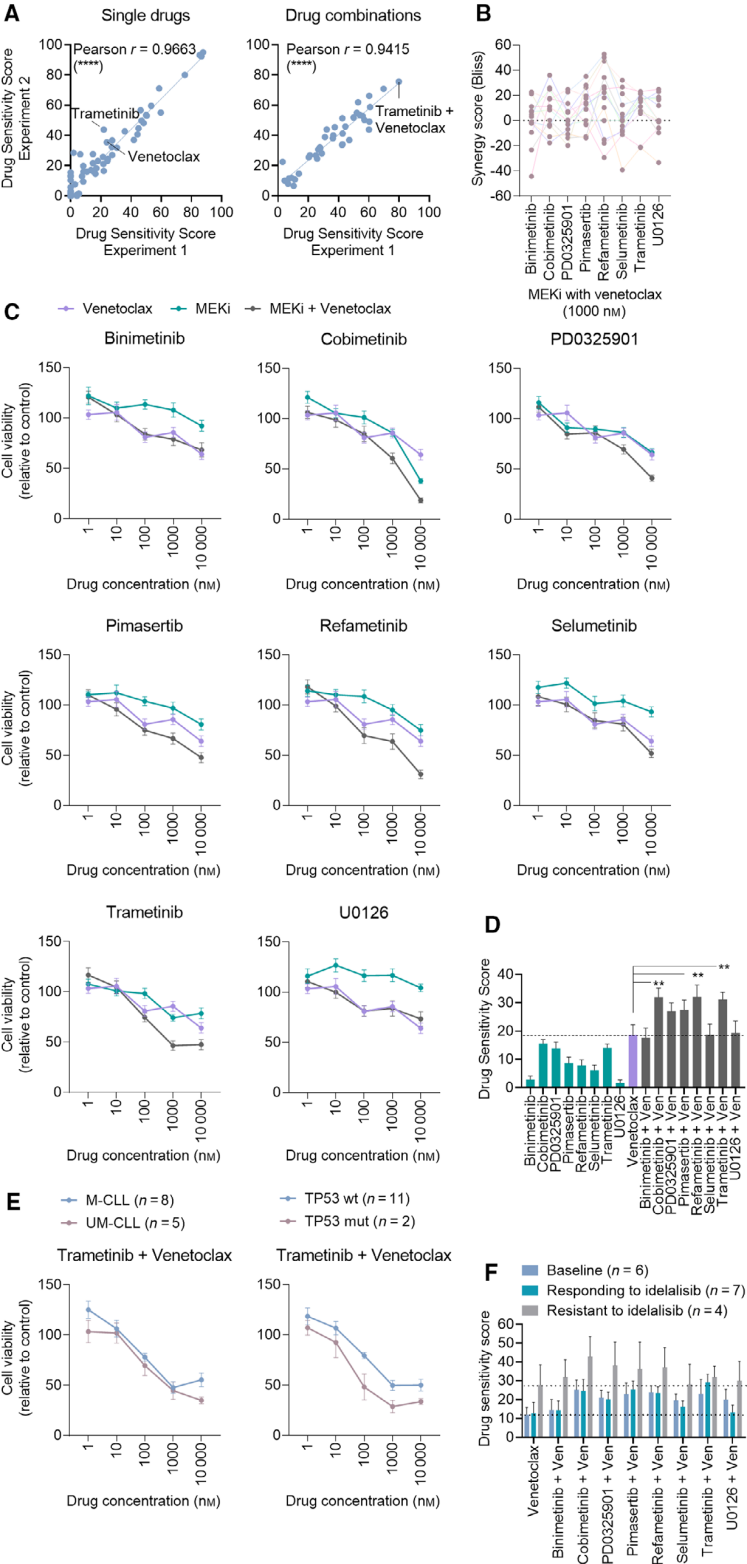
MEK/Bcl‐2 inhibition is effective in CLL independently of IGVH or TP53 mutational status and treatment history. (A) Freshly thawed OSU‐CLL cells were seeded out in 384‐well plates preprinted with a custom‐made drug library of 71 single drugs and 39 drug combinations. Cell viability was measured after 72 h by CellTiter‐Glo. DSS were calculated based on the area under the concentration–response curve (see Materials and Methods), and Pearson’s correlation analyses of results from two independent experiments were performed. ****P (two‐tailed) < 0.0001. (B) Peripheral blood mononuclear cells (PBMCs) from CLL patients (*n* = 13) were cocultured with CD40L+, BAFF+, and APRIL+ L cells (ratio 1 : 1 : 1) for 24 h prior to the initiation of the experiment to mimic the tumor microenvironment. The L cells were then removed, and the CLL cells were treated as indicated with MEKi + venetoclax (1000 nm of each drug) combinations for 72 h. Cell viability was assessed with CellTiter‐Glo. The graph shows Bliss synergy scores. Colored lines connect data‐points collected from the same patient sample. (C) As in (B), but graphs show mean relative cell viability. Error bars indicate standard error of the mean (SEM). (D) DSS were calculated for the experiments performed in (B) based on the area under the concentration–response curve (Materials and Methods). The graph shows mean DSS for the indicated treatments. Error bars indicate SEM. Statistics were performed using a 2‐way ANOVA with Tukey’s multiple comparisons test. **P < 0.005. (E) The experiments performed in (B) were stratified for the prognostic markers immunoglobulin heavy‐chain gene variable region (IGVH) mutational status (M‐CLL; mutated, UM‐CLL; unmutated) and tumor protein p53 (TP53) mutation (wt; wild‐type, mut; mutated). Graphs show mean relative cell viability. Error bars indicate SEM. (F) Drug sensitivity screens were performed on PBMCs collected from CLL patients before the patients started treatment with idelalisib (baseline; *n* = 6), while the patients were responding to idelalisib (*n* = 7), and when the patients had become resistant to idelalisib (*n* = 4). The graph shows mean DSS. Error bars indicate SEM.

To identify novel drug sensitivities and synergies in CLL, we performed drug sensitivity screens with a custom‐made drug library consisting of both single agents and drug combinations (Tables [Link], [Link]). The reproducibility of the assay was indicated by significant correlation between the DSS achieved in two independent screens on the CLL cell line OSU‐CLL (Pearson’s *r* = 0.94–0.97, *P* < 0.00001; Fig. 1A). In these screens, combined treatment with the MEKi trametinib and the Bcl‐2 antagonist venetoclax came out as most effective (Figs 1A and Fig. S1).

To verify this finding, we next studied the responses to MEKi/Bcl‐2i treatment in primary CLL cells. The drug library was expanded with 7 additional MEKi/Bcl‐2i combinations (Table S2) and screened on PBMCs from 13 CLL patient samples. We observed synergy between all eight MEKi/Bcl‐2i combinations (Fig. 1B, Table 1). While variation in responses was detected between patient samples, at least one MEKi/Bcl‐2i combination behaved synergistically in each patient sample (see color‐coded connecting lines in Fig. 1B). The effect of the individual drug combinations on relative cell viability is shown in Fig. 1C. When the concentration–response relationships were summarized using the DSS (Fig. 1D), combined treatment with venetoclax and cobimetinib, refametinib, or trametinib resulted in a statistically significant higher mean DSS relative to treatment with venetoclax single agent (mean DSS ± SEM = 32.0 ± 3.2, 32.1 ± 4.3, 31.2 ± 2.5 versus mean DSS ± SEM = 18.7 ± 3.5, respectively) (Fig. 1D). Treatment with several of the MEKi as single agents also resulted in high DSS, in particular with cobimetinib (mean DSS ± SEM = 15.57 ± 1.46), PD0325901 (mean DSS ± SEM = 13.95 ± 2.15), and trametinib (mean DSS ± SEM = 14.13 ± 1.33). These findings suggest that MEKi are active in CLL, and combined MEK/Bcl‐2 inhibition has synergistic potential.

### 3.2 Sensitivity to MEK/Bcl‐2 inhibition is independent of high‐risk features and CLL treatment status

To study the impact of high‐risk features on sensitivity to MEK/Bcl‐2 inhibition, we stratified the patient samples according to IGVH and TP53 mutational status. We found that combination treatment was equally effective in all patient subgroups, with a trend toward increased sensitivity in TP53‐mutated CLL cells (Fig. 1E and not shown). Although the number of patients included in this study was relatively small, these findings indicate that also high‐risk CLL may benefit from combined MEK/Bcl‐2 inhibition.

Most of the patients included in this study had never received treatment for CLL (10/13 patients; ‘CLL’ patients listed in Table 2). To investigate whether treatment status affected sensitivity to MEK/Bcl‐2 inhibition, we performed the same analysis on a cohort of CLL patients that were treated with the PI3K inhibitor idelalisib as first line or following chemoimmunotherapy (‘JB’ patients listed in Table 2) [8]. Drug sensitivity screens were performed on the patients’ CLL cells collected prior to start of treatment with idelalisib (*n* = 6), while the patients were responding to idelalisib (*n* = 7), and when the patients had developed resistance to idelalisib (*n* = 4) (Fig. 1F). We found that the cells were responsive to treatment at all time‐points, with a trend toward increased sensitivity in CLL cells collected from idelalisib‐resistant patients (Fig. 1F, gray bars). Taken together, these findings indicate that MEKi/Bcl‐2i are effective in CLL cells from treatment‐naïve, treated, and treatment‐resistant patients.

### 3.3 MEK/Bcl‐2 inhibition shows selectivity for CLL cells, and early induction of apoptosis predicts sensitivity

**Fig. 2:**
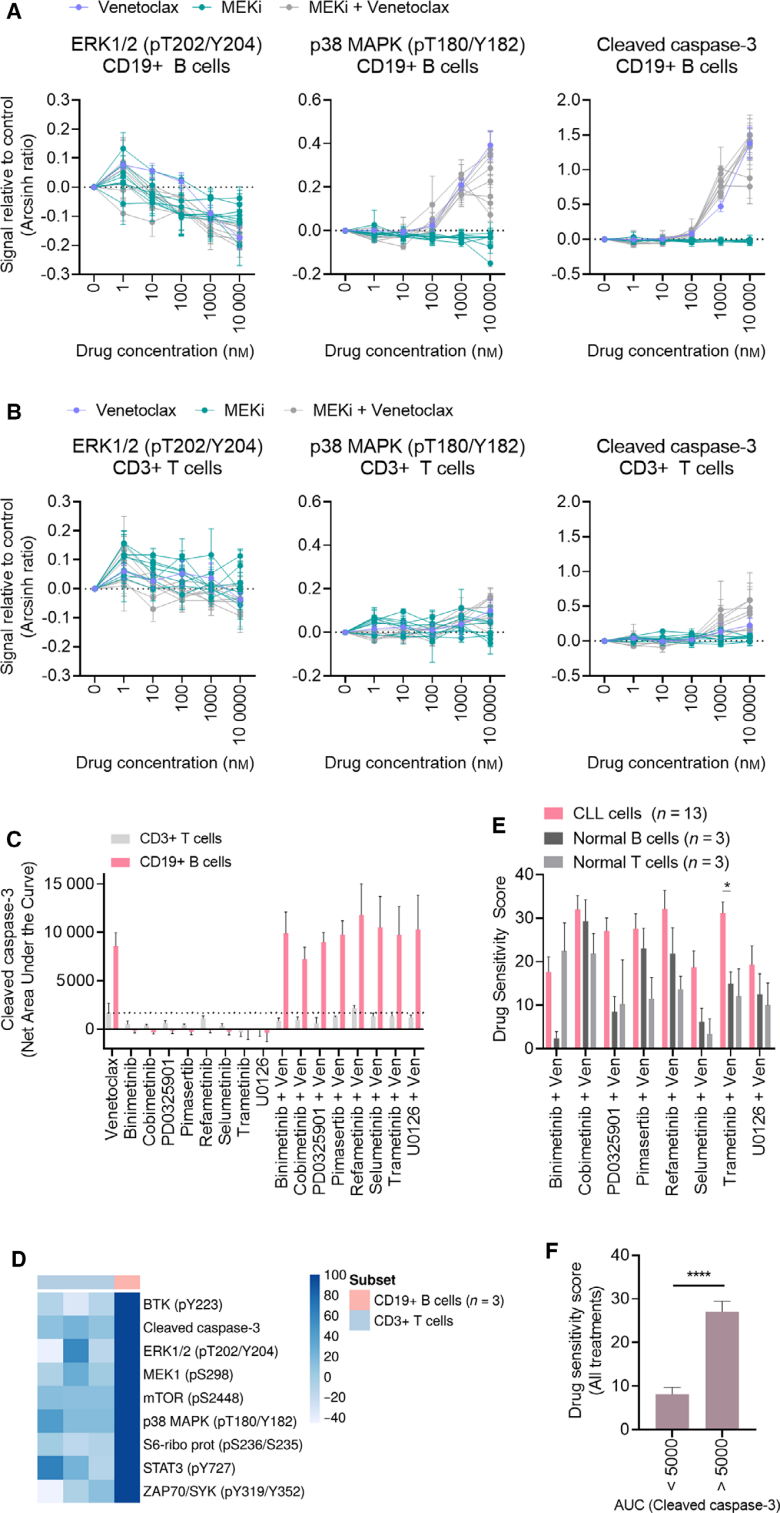
MEK/Bcl‐2 inhibition is selective for CD19^+^ CLL cells, and early induction of apoptosis predicts sensitivity. (A) PBMCs from CLL patients (*n* = 3) were treated with 8 MEKi (Table 1) alone or in combination with venetoclax as indicated for 30 min, followed by 5‐min anti‐IgM stimulation. The cells were then fixed, permeabilized, and stained with the indicated antibodies. Signals were analyzed by flow cytometry. Results are shown for CD19^+^ cells. Raw data were transformed to an arcsinh ratio relative to the signal in dimethyl sulfoxide (DMSO)‐treated control cells, which were set to zero. Curves show the mean of the three experiments. Error bars indicate SEM. (B) As in (A), but results are shown for CD3^+^ T cells. (C) Net AUC was calculated from the drug response curves of cleaved caspase‐3 shown in (A and B). Error bars indicate SEM. (D) Net AUC values were calculated from the trametinib + venetoclax drug response curves of the indicated proteins (rows), collected in the experiments shown in (A and B). The net AUC values of the CD3^+^ populations were then normalized to the mean of the corresponding CD19^+^ populations (*n* = 3), which was set to 100. The heatmap was created using the normalized AUC values as input in the online tool ClustVis (https://biit.cs.ut.ee/clustvis/). (E) Drug sensitivity testing was performed with the indicated drug combinations on PBMCs from CLL patients (*n* = 13) and on CD19^+^ B cells and CD3^+^ T cells isolated from healthy blood donors (*n* = 3). Cell viability was measured after 72 h by CellTiter‐Glo. The graph shows mean DSS for the indicated treatments. Error bars indicate SEM. Statistics were performed using a 2‐way ANOVA with Tukey’s multiple comparisons test. *P < 0.05. (F) Treatment responses were grouped based on the net AUC calculated from the response curves of cleaved caspase‐3 shown in (A) (< 5000 and > 5000), and plotted against the respective DSS shown in (E). Error bars indicate SEM. Statistics were performed using a paired *t*‐test. ****P < 0.0001.

We next screened for early effects of drug treatment on a panel of intracellular proteins. PBMCs from three CLL patient samples were treated with single agents or drug combinations for 30 min, followed by 5‐min stimulation with anti‐IgM to activate the B‐cell receptor (Fig. 2A,B). As expected, we found that MEK inhibition resulted in a concentration‐dependent reduction in ERK1/2 (pT202/Y204), a downstream substrate of MEK, in CD19^+^ CLL cells (Fig. 2A, left panel, green curves). We also observed that treatment with venetoclax, but not with MEKi single agents, induced activation of p38 MAPK (pT180/Y182) (Fig. 2A, middle panel, purple curve). Venetoclax exposure further induced apoptosis in this system, as shown by elevation of cleaved caspase‐3 (Fig. 2A, right panel, purple curve). This effect was enhanced with combined MEK/Bcl‐2 inhibition (Fig. 2A, right panel, gray curves). It has been reported that activation of p38 MAPK can suppress ERK1/2 activity during induction of apoptosis [33]. In agreement with this, we found that venetoclax exposure induced inhibition of ERK1/2 (pT202/Y204) at the same concentrations (> 100 nm) that resulted in p38 MAPK activation and cleavage of caspase‐3 (Fig. 2A, left panel, purple curve). Taken together, these results demonstrate that both MEKi and venetoclax, directly or indirectly, inhibit ERK1/2 signaling, and that effects of MEK/Bcl‐2 inhibition can be detected early—already after 30 min of drug exposure.

To study the selectivity of the treatments, we gated the CLL PBMCs on CD3^+^ T cells in the same experiments (Fig. 2B). While the CD19^+^ B cells became stimulated by anti‐IgM, the CD3^+^ T cells remained unaffected, which may affect signaling amplitudes. Regardless, the drug‐induced changes in cell signaling and apoptosis were minor in T cells compared with B cells, indicating a certain degree of selectivity of the treatments toward CLL cells (Fig. 2A–D).

To further examine the selective efficacy of the treatments, we performed drug sensitivity screens on CD19^+^ B cells and CD3^+^ T cells from three healthy blood donors (Fig. 2E). We observed that most combination treatments were more effective in CLL cells than in normal B or T cells, and the difference reached statistical significance for trametinib + venetoclax (Fig. 2E).

We next investigated whether the induction of apoptosis observed after only 30 min of drug exposure (Fig. 2A) could predict drug sensitivity in 72‐h assays (Fig. 1D). To this end, we calculated the net area under the curve (AUC) of the concentration–response curves for cleaved caspase‐3. We observed that the treatments resulted in distinct responses, which could be grouped as AUC < 5000 and AUC > 5000 (Fig. 2F). Treatment with venetoclax alone or in combination with a MEKi resulted in an AUC > 5000 (Fig. 2C). The mean DSS in the AUC < 5000 group was significantly lower than the mean DSS in the AUC > 5000 group, suggesting a positive correlation between early induction of apoptosis and drug sensitivity (Fig. 2F).

Taken together, these findings indicate that MEK/Bcl‐2 inhibition shows selectivity for CLL cells, and that early induction of apoptosis can predict sensitivity in 72‐h assays. While the clinical utility of our findings needs to be tested, a biomarker for drug sensitivity that can be detected within a few hours could prove useful to guide treatment decisions for patients in urgent need of therapy.

### 3.4 MEK inhibition induces cell death of CLL and MM, but not of MCL

**Fig. 3:**
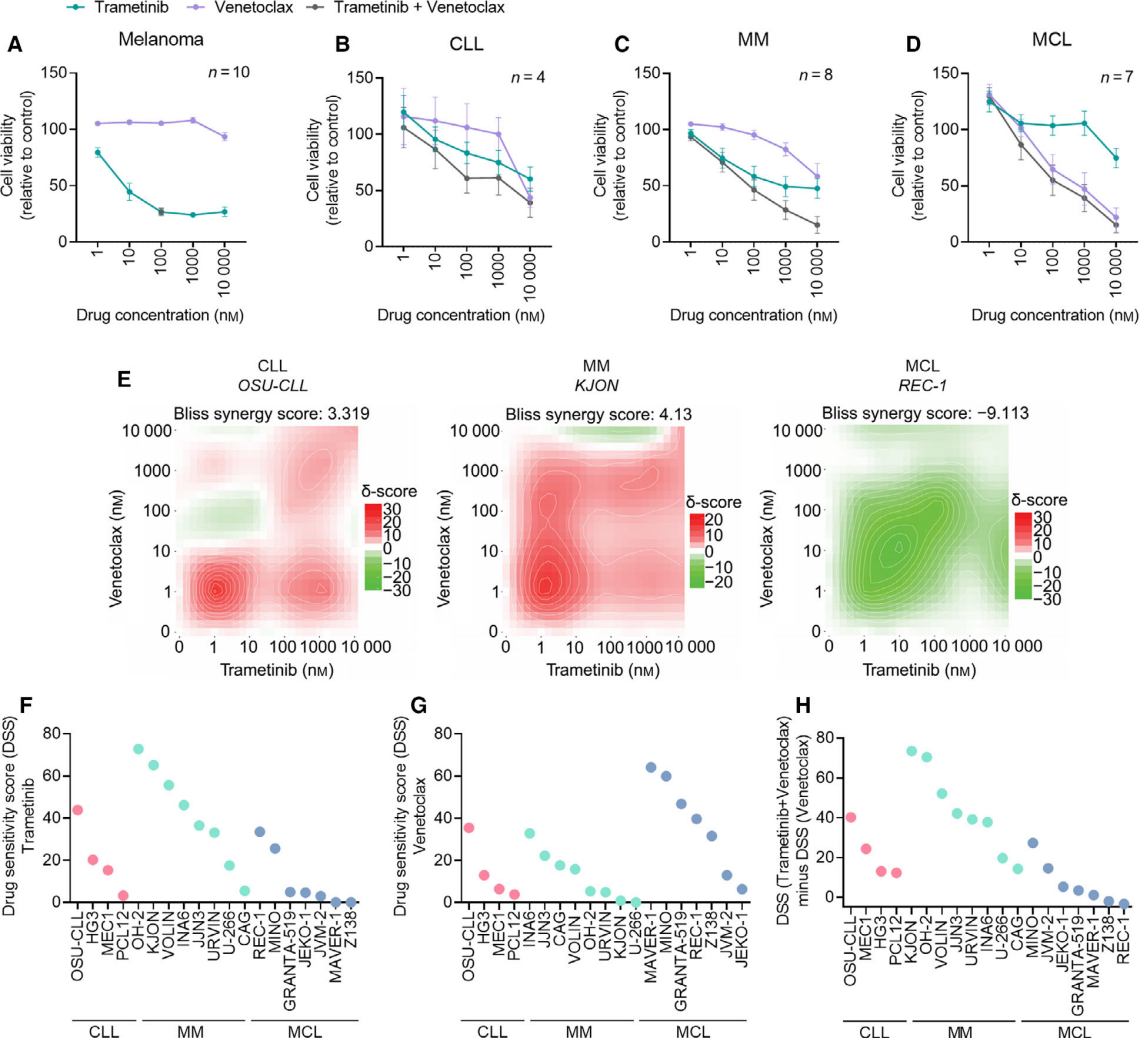
MEK inhibition induces cell death of CLL and MM, but not of MCL. (A) Drug sensitivity to the indicated treatments was assessed on *n* = 10 melanoma cell lines after 72‐h exposure using CellTiter‐Glo. The combination was tested at 100 nm. The graph shows relative cell viability. Error bars indicate SEM. (B) Drug sensitivity to the indicated treatments was assessed on *n* = 4 CLL cell lines after 72‐h exposure using CellTiter‐Glo. The graph shows relative cell viability. Error bars indicate SEM. (C) as in (B), but on *n* = 8 MM cell lines. (D) as in (B), but on *n* = 7 MCL cell lines. (E) The concentration–response data from drug sensitivity screens on CLL, MM, and MCL cell lines were processed through the DECREASE tool to predict the full drug combination concentration–response matrices. The data were then provided as input to SynergyFinder, which calculates the synergy. One representative plot is shown for each disease model. (F) DSS for trametinib treatment was calculated from the experiments in (B–D). (G) DSS for venetoclax treatment was calculated from the experiments in (B–D). (H) Selective DSS to trametinib + venetoclax treatment was calculated by subtracting DSS (venetoclax) from DSS (venetoclax + trametinib). Results are based on the experiments in (B–D).

To study whether the observed sensitivity to MEK/Bcl‐2 inhibition was specific to CLL or a more general feature of B‐cell malignancies, we expanded the study to cell line models of CLL (*n* = 4), MM (*n* = 8), and MCL (*n* = 7). Melanoma cell lines (*n* = 10) were used as a positive control for sensitivity to MEKi, as this drug class has been approved for treatment of this disease (Table 1). Here, trametinib was used as a representative MEKi as it was effective in CLL and is approved by the European Medicines Agency (EMA) and FDA (Table 1). As shown, the melanoma cell lines were responsive to trametinib, but not to venetoclax (Fig. 3A). Furthermore, no synergy was observed between trametinib and venetoclax in the melanoma lines at 100 nm concentrations (see gray point in Fig. 3A).

Drug sensitivity screens on the different cell line models showed that CLL and MM cells were responsive to trametinib (Fig. 3B,C), and there was synergy between trametinib and venetoclax (Fig. 3E, left and middle panels). However, the MCL cell lines did not respond to trametinib treatment (Fig. 3D), and there was no synergy between venetoclax and trametinib in this model (Fig. 3E, right panel). The DSS of the individual cell lines showed that MM cell lines were most sensitive to trametinib (Fig. 3F) and the combination (Fig. 3H), while MCL cell lines were more responsive to venetoclax (Fig. 3G).

In general, moderate heterogeneity in drug sensitivity was observed between cell lines of the same disease origin. Among the CLL cell lines, OSU‐CLL cells were most sensitive to the treatments, while PCL12 cells were less sensitive (Fig. 3F–H). OSU‐CLL has mutated IGVH, trisomy 12, trisomy 19, noncomplex karyotype, and wild‐type p53 [15], while PCL12 has unmutated IGVH and no cytogenetic or genetic lesions [16]. With the small number of analyzed CLL cell lines (*n* = 4), it was not possible to stratify them based on such molecular features. However, the impact of IGVH and TP53 mutational status on sensitivity to trametinib + venetoclax in primary CLL cells was investigated in Fig. 1E. While this cohort was also moderate (*n* = 13), the results suggest that high‐risk features do not negatively affect sensitivity to trametinib + venetoclax. Together, these findings suggest differences in MEKi/Bcl‐2i sensitivities across B‐cell malignancies.

### 3.5 Intracellular protein profiling of CLL, MM, and MCL cells

**Fig. 4:**
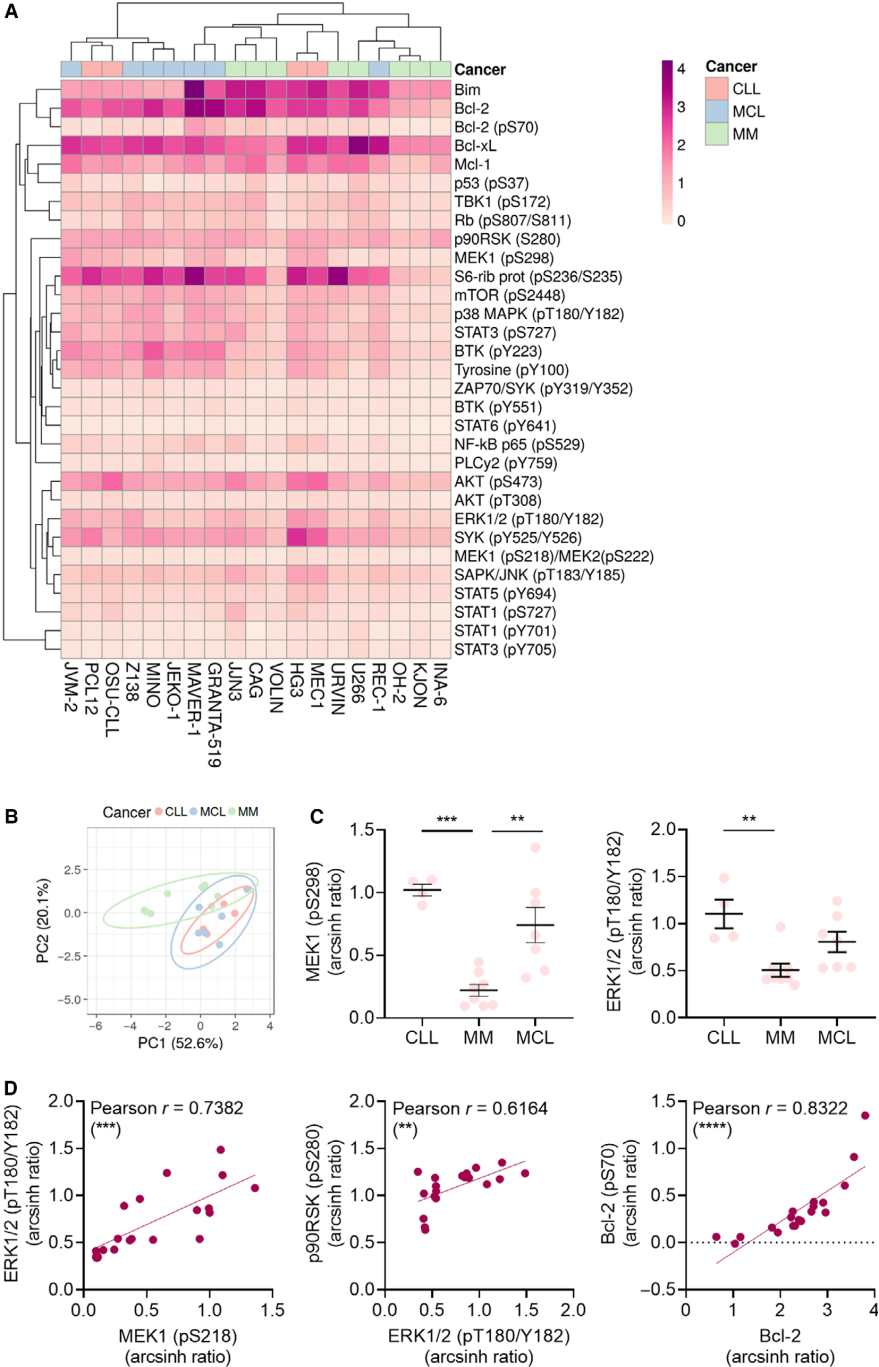
Intracellular protein profiling of CLL, MM, and MCL. (A) Freshly thawed CLL (*n* = 4), MM (*n* = 8), and MCL (*n* = 7) cell lines (columns) were fixed, permeabilized, and stained with antibodies against the indicated proteins (rows). Signals were analyzed by flow cytometry. Raw data were transformed to an arcsinh ratio relative to the signal of an isotype control, which was set to zero. The heatmap was created using ClustVis (https://biit.cs.ut.ee/clustvis/). Both rows and columns are clustered using correlation distance and average linkage. (B) As in (A), but raw data were used to calculate principal components (PCs) in ClustVis (https://biit.cs.ut.ee/clustvis/). No scaling was applied to rows. The *x*‐ and *y*‐axes show PC1 and PC2 that explain 52.6% and 20.1% of the total variance, respectively. Prediction ellipses show 95% confidence interval. (C) The levels of MEK (pS298) and ERK1/2 (pT180/Y182) detected in (A) are shown for CLL, MM, and MCL cell lines. Each data point represents one cell line. Bars indicate mean ± SEM. Statistical testing was done using the one‐way ANOVA with Tukey’s multiple comparisons test. **P < 0.005, ***P < 0.001. (D) Pearson’s correlation analyses were performed on the indicated protein levels detected in (A). Each data point represents one cell line. **P (two‐tailed) < 0.005, ***P (two‐tailed) < 0.001, and ****P (two‐tailed) < 0.0001.

To gain a mechanistic understanding of the differences in drug sensitivities between cell line models, we performed a high‐throughput profiling of 31 intracellular proteins by phospho flow cytometry across the 19 CLL, MM, and MCL cell lines (Fig. 4A). Protein levels varied between cell lines, but appeared to be similar within disease models as shown by principal component analysis (Fig. 4B). The CLL and MCL cell lines showed overlapping profiles, while the MM cell lines formed a more separate cluster (Fig. 4B). This may be explained by a general trend toward lower protein expression and activation in these cell lines (Fig. 4A), as illustrated by the low levels of MEK1 (pS298) and ERK1/2 (pT180/Y182; Fig. 4C).

As expected, we observed a statistically significant correlation between phosphorylation levels of proteins in the same pathway, including MEK1 (pS218) and its substrate ERK1/2 (pT180/Y182; Fig. 4D), and between ERK1/2 (pT180/Y182) and its substrate p90RSK (pS280; Fig. 4D). We also observed a statistically significant correlation between the phosphorylated and nonphosphorylated versions of Bcl‐2 (Fig. 4D).

### 3.6 Protein expression level and activation status correlate with drug responsiveness

**Fig. 5:**
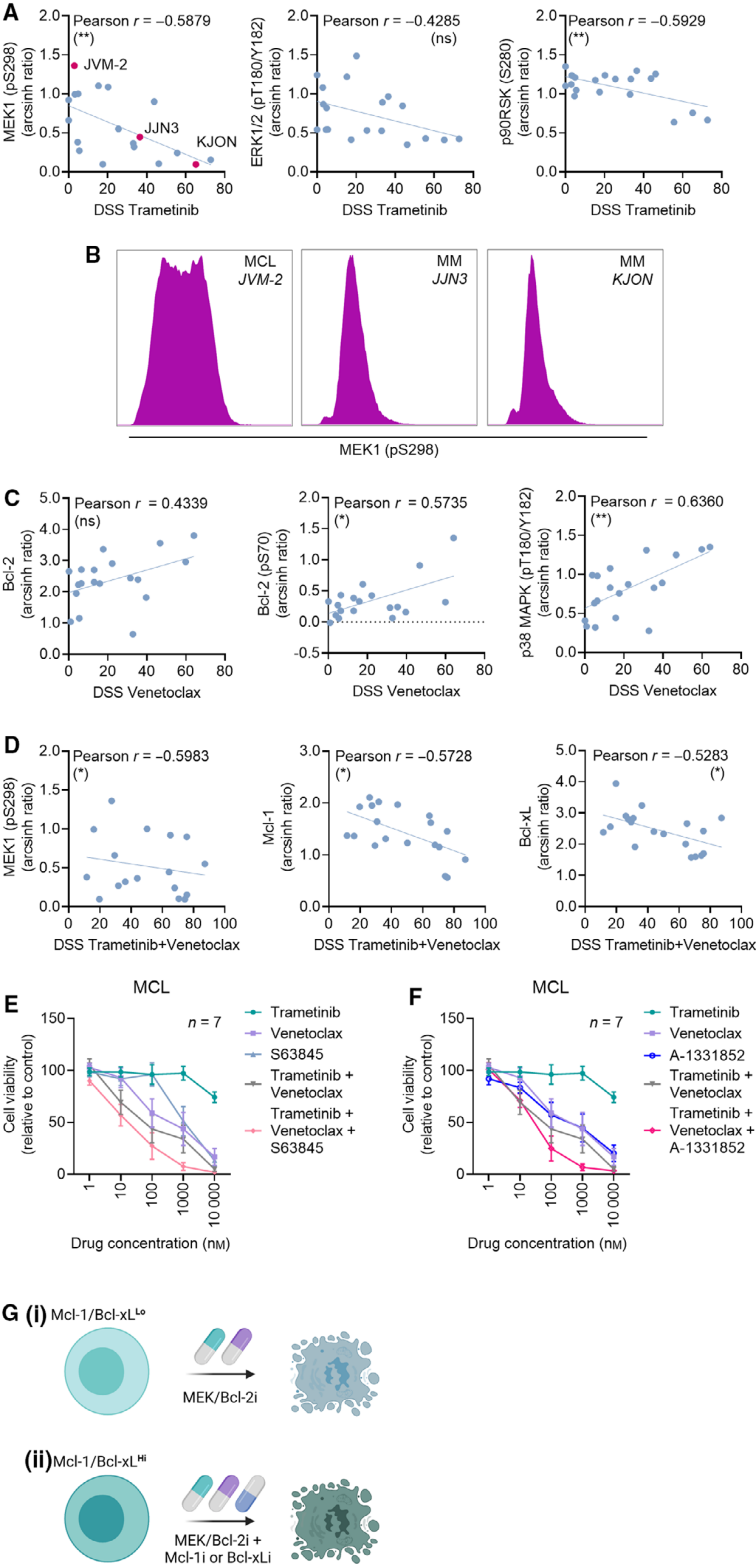
Expression level of Mcl‐1 and Bcl‐xL predicts MEKi/Bcl‐2i vulnerability. (A) Pearson’s correlation analyses were performed based on the relative protein levels detected in Fig. 4A and DSS to trametinib in *n* = 19 cell lines. Three cell lines with low, intermediate, and high levels of MEK1 (pS298) are highlighted in purple. **P (two‐tailed) < 0.005, ns (not significant). (B) Histograms show MEK1 (pS298) signals in three representative cell lines with high (JVM‐2), intermediate (JJN3), and low (KJON) levels. The histograms were created using Cytobank (https://cellmass.cytobank.org/cytobank/). (C) As in (A), but for DSS to venetoclax. *P (two‐tailed) < 0.05, **P (two‐tailed) < 0.005, ns (not significant). (D) As in (A), but for DSS to trametinib + venetoclax. *P (two‐tailed) < 0.05. (E) Drug sensitivity to the indicated treatments was assessed on *n* = 7 MCL cell lines after 72‐h exposure using CellTiter‐Glo. The graph shows relative cell viability. Error bars indicate SEM. (F) As in (E), but the Bcl‐xL inhibitor A‐1331852 was used instead of the Mcl‐1 inhibitor S63845. The graph shows relative cell viability. Error bars indicate SEM. (G) Illustration of effective combinatorial strategies for B‐cell malignancies with (i) low expression of Mcl‐1/Bcl‐xL, or (ii) high expression of Mcl‐1/Bcl‐xL.

To investigate whether protein expression level or activation status could predict drug sensitivities, we performed correlation analyses between protein readouts and DSS for trametinib, venetoclax, and the combination (Fig. 5A–D). We found that the DSS for trametinib inversely correlated with basal phosphorylation levels of MEK1 (*r* = −0.59, *P* < 0.01), and the downstream proteins ERK1/2 (*r* = −0.43, n.s.) and p90RSK (*r* = −0.59, *P* < 0.01; Fig. 5A,B). This is in line with an earlier study showing that the efficacy of the SYK inhibitor fostamatinib is lower in MCL cells with high phospho‐SYK levels than in MCL cells with low phospho‐SYK levels [34]. On the contrary, we found that high expression of Bcl‐2 and phosphorylated Bcl‐2 positively correlated with venetoclax sensitivity (*r* = 0.43, n.s. and *r* = 0.57, *P* < 0.05, respectively; Fig. 5C). Similarly, high phosphorylation levels of p38 MAPK correlated with high responsiveness to venetoclax (*r* = 0.64, *P* < 0.01; Fig. 5C). This is of interest as we found that exposure to venetoclax induces p38 MAPK phosphorylation (Fig. 2A), suggesting a positive feedback loop.

Similar to its predictive value for trametinib sensitivity, MEK1 phosphorylation levels inversely correlated with DSS to trametinib + venetoclax as well (Fig. 5D). In addition, expression levels of the antiapoptotic Bcl‐2 family members Mcl‐1 and Bcl‐xL showed significant inverse correlation with DSS to trametinib + venetoclax (*r* = −0.57, *P* < 0.05, and *r* = −0.53, *P* < 0.05, respectively; Fig. 5D). While upregulation of these proteins is associated with resistance to venetoclax [1], and combined Bcl‐2/Mcl‐1 inhibition has proven effective in preclinical models [35, 36], we found that Mcl‐1 and Bcl‐xL levels also correlated with DSS to trametinib single agent (*r* = −0.52, *P* < 0.05, and *r* = −0.49, *P* < 0.05, respectively). Taken together, these findings suggest that protein expression and activation status play a role in *in vitro* drug responsiveness.

### 3.7 Low sensitivity to MEKi/Bcl‐2i in Mcl‐1/Bcl‐xL^Hi^ cells can be overcome with an Mcl‐1 or Bcl‐xL inhibitor

To test whether Mcl‐1 inhibition could sensitize cells to MEK/Bcl‐2 inhibitors, the seven MCL cell lines that were unresponsive to trametinib were used as a model. The cells were treated with trametinib, venetoclax, or the Mcl‐1 inhibitor S63845 single agents, and trametinib + venetoclax with or without S63845 (Fig. 5E). As shown, the cells responded to S63845 (Fig. 5E, blue curve), and addition of S63845 to trametinib + venetoclax potentiated the effect of the treatment on cell killing (Fig. 5E, pink curve). While the decrease in cell viability in response to the triple combination was not statistically significantly different from the response to the double combinations, a clear trend was observed, which was confirmed when the Mcl‐1 inhibitor was replaced with the Bcl‐xL inhibitor A‐1331852 (Fig. 5F). Interestingly, S63845 + venetoclax had a similar efficacy as trametinib + venetoclax on MCL cell lines (Fig. S2a, purple curve), demonstrating the independent contribution of all single agents in the more effective triple combination (Fig. S2a, pink curve). No additive effects were seen when both S63845 and A‐1331852 were added to trametinib + venetoclax, relative to the triple combinations (Fig. S2b, dark pink curve). Similarly, MEK/Mcl‐1/Bcl‐xL inhibition was less effective than MEK/Bcl‐2 + Mcl‐1 or Bcl‐xL inhibition (Fig. S2b, green curve). Similar experiments on CLL cell lines showed some additive effect of the Bcl‐xL inhibitor when combined with trametinib + venetoclax (Fig. S2c, dark pink line), while the Mcl‐1 inhibitor was ineffective in this model (Fig. S2c).

Taken together, these findings demonstrate that protein profiling can accurately identify treatment vulnerabilities, which may be explored in a clinical setting (Fig. 5G).

## 4 Discussion

Combination therapy is used as a strategy to delay treatment resistance [1]. The Bcl‐2 antagonist venetoclax is an attractive partner for therapies that target the B‐cell receptor pathway, since it targets the distinct apoptotic pathway. Drug synergy between venetoclax and inhibitors that act downstream of the B‐cell receptor is therefore likely. Several studies are currently investigating combinations between venetoclax and BTK or PI3K inhibitors in CLL, which are the most advanced targeted therapies in this disease [4]. Here, we aimed to identify novel therapeutic options for CLL and related B‐cell malignancies.

We detected synergy between MEKi and venetoclax in preclinical models of CLL and MM. Combinations between venetoclax and a MEKi have been reported to be efficacious also in other preclinical studies on hematological malignancies [12, 37, 38, 39], and these combinations are already being investigated in some hematological diseases. For instance, venetoclax + trametinib is currently studied in acute myeloid leukemia (AML) (NCT04487106; Table 1), while two other studies are investigating the effect of combining venetoclax with cobimetinib in MM and AML (NCT03312530, NCT02670044; Table 1). We showed that combined treatment with venetoclax and cobimetinib or trametinib is among the most effective MEK/Bcl‐2 combinations in CLL. These findings align well with the advanced clinical investigations of these therapies and suggest that clinical studies with these treatments are warranted also in CLL.

Of interest, we found that the efficacy of combined MEK/Bcl‐2 inhibition in primary CLL cells was independent of IGVH and TP53 mutational status. While the investigated cohort was relatively small (*n* = 13), this finding suggests that MEK/Bcl‐2 inhibition is of relevance to both low‐ and high‐risk CLL. This is in agreement with results from the Phase II CAPTIVATE study (NCT02910583), where CLL patients received frontline treatment with the targeted therapies venetoclax plus the BTK inhibitor ibrutinib [40]. In this study, high undetectable minimal residual disease (uMRD) was observed across patient subgroups, including those with high‐risk disease features [40]. Clinical data on MEK/Bcl‐2 inhibition in CLL are needed to determine in what setting this combination may be used.

MEKi are primarily indicated for BRAF+ melanoma (Table 1). Activating mutations in BRAF leads to downstream activation of MEK, thus providing a rationale for MEK inhibition. BRAF mutations are less frequent in hematological malignancies. In CLL, there was only a 2.8% occurrence in a cohort of 138 patients [41]. Such mutations are therefore unlikely to fully explain the sensitivity to MEK inhibition observed here. Furthermore, it has been shown that activation of ERK is associated with resistance to idelalisib in CLL irrespective of mutations in the MAPK pathway [8]. To investigate underlying mechanisms of the disease‐specific drug sensitivities observed here, we performed high‐throughput profiling of 31 intracellular proteins, including members of the MAPK signaling pathway. We found that high basal phosphorylation level of MAPK pathway proteins correlated with low sensitivity to trametinib in CLL, MM, and MCL cell lines. This is in line with a previous study showing that increased doses of the SYK inhibitor fostamatinib are required to overcome high phospho‐SYK levels in MCL [34].

Interestingly, we showed that CLL cells from idelalisib‐resistant patients were sensitive to dual MEK/Bcl‐2 inhibition. Three of these patients (JB‐0058, JB‐0157, and JB‐0158) were included in an earlier study that identified activating mutations in MAPK pathway genes as a mechanism of idelalisib resistance [8]. However, the three commonly analyzed patients did not have such mutations [8]. In the reported study, combination therapy with PI3K/ERK inhibitors was suggested as a strategy to overcome PI3Ki resistance [8]. The findings herein underscore the potential of targeting MEK in idelalisib‐resistant CLL.

Low sensitivity to dual MEK/Bcl‐2 inhibition observed in some cell lines, particularly those of MCL origin, could be explained by high expression levels of the antiapoptotic Bcl‐2 family proteins Mcl‐1 or Bcl‐xL. Interestingly, an shRNA drug screen previously identified Bcl‐xL as a target for combination therapy with MEKi in different cancer models [42], and high expression of Bcl‐xL is associated with resistance to chemotherapy in B‐cell acute lymphoblastic leukemia (B‐ALL) [43]. Bcl‐xL binds and inhibits the proapoptotic protein BIM, which was shown to be upregulated in response to MEK inhibition [44]. We showed that adding a Bcl‐xL or Mcl‐1 inhibitor to trametinib + venetoclax enhanced the efficacy of the combination in MCL. Similarly, Mcl‐1 inhibition has been shown to enhance the efficacy of MEKi in lung cancer [45]. This efficacy was further amplified by prior exposure to Bcl‐xL inhibitors. Combined MEK and Bcl‐2/xL inhibition was also shown to be effective in high‐grade serous ovarian cancer patient‐derived xenograft models [46]. A phase I/II study of combined MEK and Bcl‐xL inhibition has shown initial signs of efficacy, with a favorable disease control rate and durable partial response in patients with RAS mutant gynecological cancer [47], demonstrating the clinical utility of the treatment strategy.

Our findings suggest that Mcl‐1 and Bcl‐xL expression levels predict responsiveness to MEK/Bcl‐2 inhibition. Cells with high expression of Mcl‐1 or Bcl‐xL and low responsiveness to MEK/Bcl‐2 inhibition remained sensitive to inhibitors targeting these antiapoptotic proteins. In these cases, a triple combination was indeed effective (Fig. 5G). Our *in vitro* study thus indicates that Mcl‐1 and Bcl‐xL may serve as biomarkers to guide treatment decisions. However, additional preclinical studies are required to validate these findings and to determine the threshold that defines treatment responsiveness. Of note, we studied drug responsiveness in CLL cells from peripheral blood. Since it has been reported that the expression level of Bcl‐xL is higher in lymph nodes than in peripheral blood in CLL patients [48], it will be of interest to determine how this impacts drug sensitivity to MEK/Bcl‐2 inhibition *in vivo*. This will be the focus of future studies.

Taken together, we provide preclinical evidence for biomarker‐guided combinatorial strategies targeting MEK and Bcl‐2 family members in B‐cell malignancies.

## 5 Conclusions

Our study showed that combined treatment with a MEKi and the Bcl‐2 antagonist venetoclax was effective in CLL cells, independently of high‐risk prognostic markers. The combination was effective also in MM, but not in MCL cells. High expression of Mcl‐1 or Bcl‐xL predicted the low sensitivity, which could be overcome by adding in an Mcl‐1 or Bcl‐xL inhibitor.

## Conflict of interest

JRB has served as a consultant for AbbVie, Acerta/AstraZeneca, BeiGene, Bristol‐Myers Squibb/Juno/Celgene, Catapult, Genentech/Roche, Eli Lilly, Janssen, MEI Pharma, MorphoSys AG, Nextcea, Novartis, Pfizer, and Rigel; received research funding from Gilead, Loxo/Lilly, Verastem/Secura Bio, Sun, and TG Therapeutics; and served on the data safety monitoring committee for Invectys. The other authors declare no competing financial interests.

## Author contributions

SSS designed the research. KM, MG, LK, PA‐D, RH, and SSS performed the experiments and analyzed the data with input from JE and KT. GET and JB contributed with patient samples. TH contributed with cell lines. SSS wrote the article with KM. All authors read and commented on draft versions of the manuscript and approved the final version.

### Peer review

The peer review history for this article is available at https://publons.com/publon/10.1002/1878‐0261.13153.

## Supporting information


**Fig S1.** Drug sensitivity in OSU‐CLL.

**Fig S2.** Sensitivity to MEK/Bcl‐2/Mcl‐1/Bcl‐xL inhibition in MCL and CLL.

**Table S1.** Drug library.

**Table S2.** Drug combinations.

## Data Availability

The data that support the findings of this study are available from the corresponding author [sigrid.skanland@ous-research.no] upon reasonable request.
