# Supplementary figures and images for: Mcl‐1 and Bcl‐xL levels predict responsiveness to dual MEK/Bcl‐2 inhibition in B‐cell malignancies
Source: Mol Oncol. 2021 Dec 18;16(5):1153–70. doi: 10.1002/1878-0261.13153 (PMC8895453; doi:10.1002/1878-0261.13153)

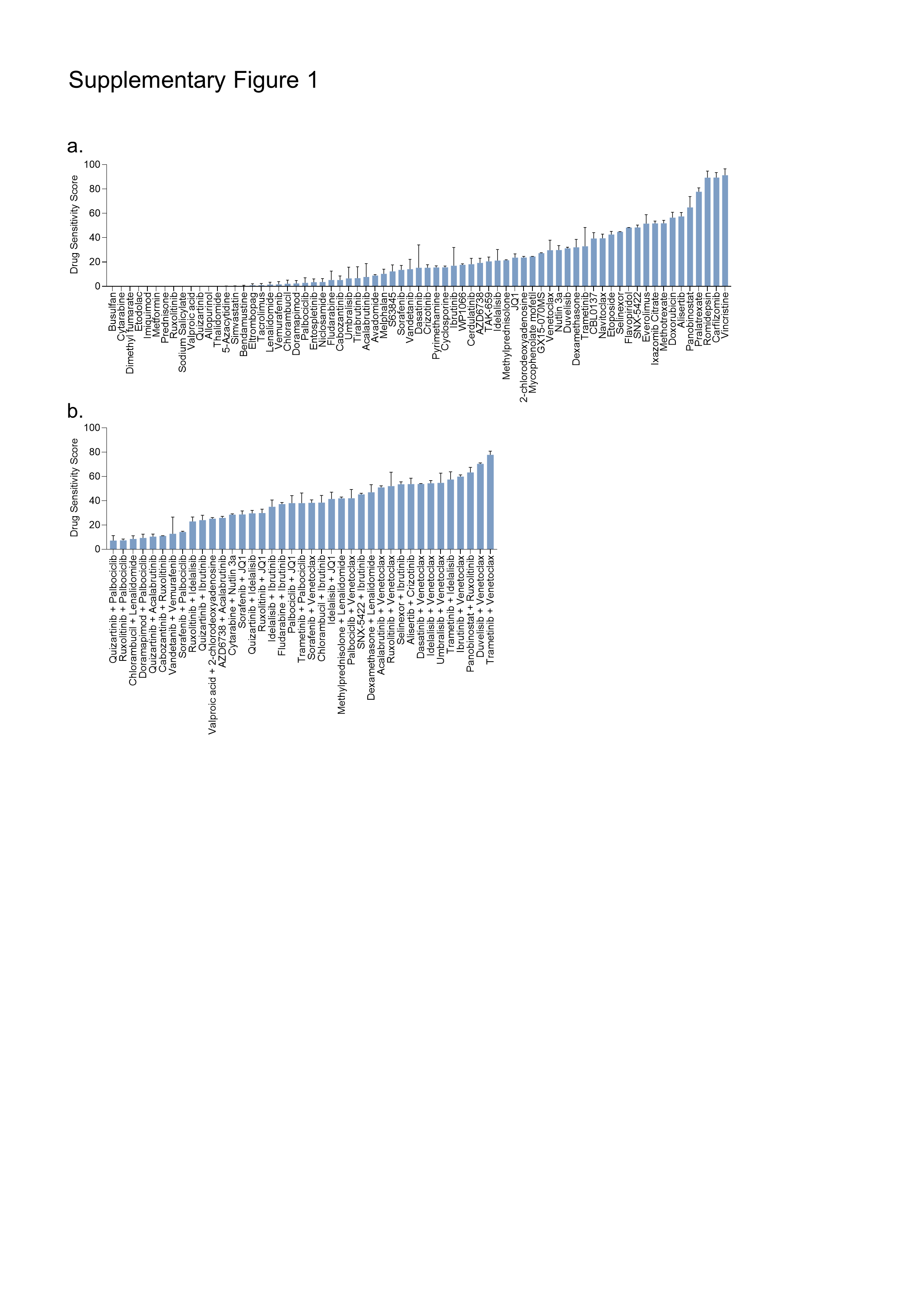

Supplement: Supplementary file 1 — Fig S1. Drug sensitivity in OSU‐CLL. [file MOL2-16-1153-s003.tif]

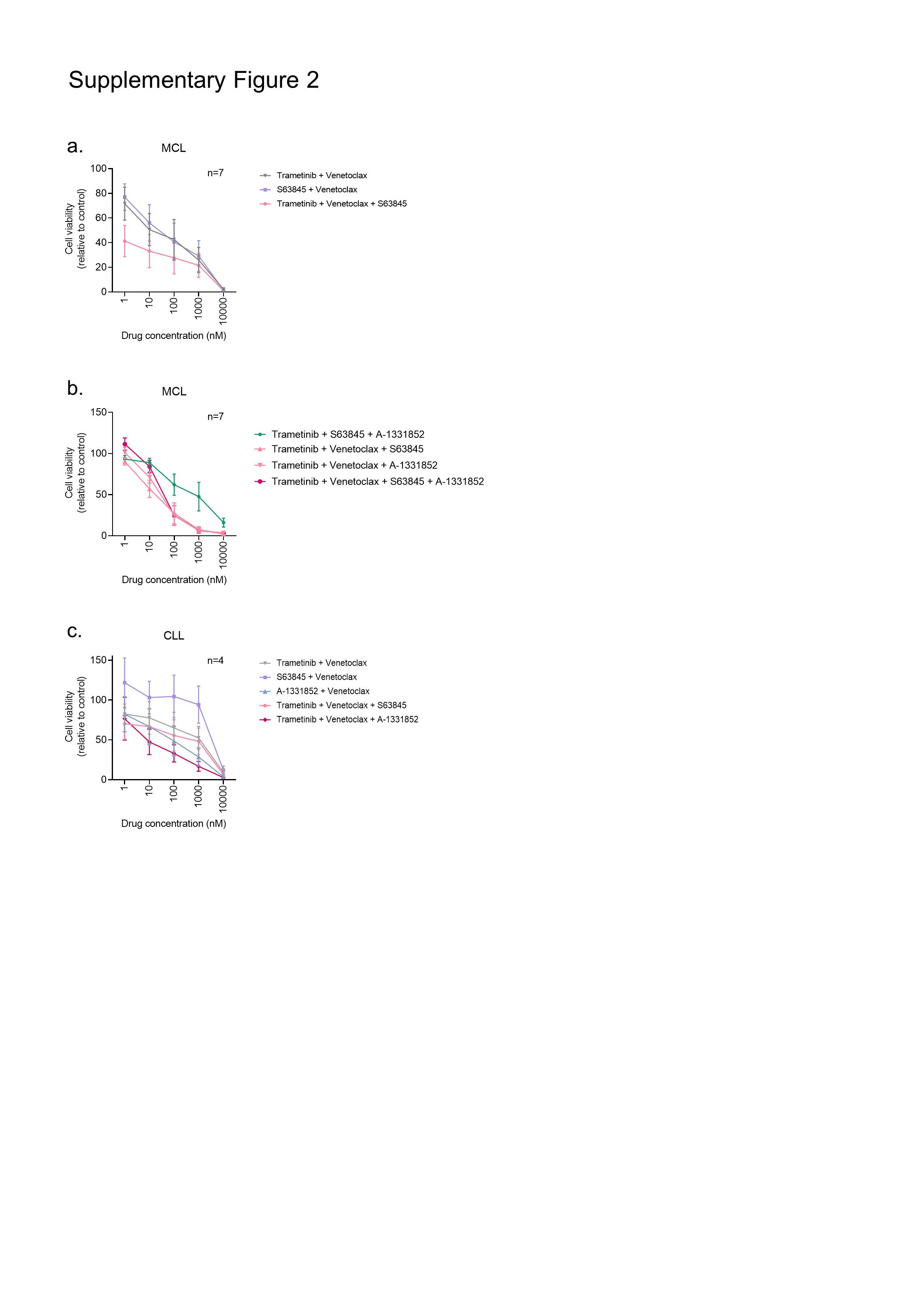

Supplement: Supplementary file 2 — Fig S2. Sensitivity to MEK/Bcl‐2/Mcl‐1/Bcl‐xL inhibition in MCL and CLL. [file MOL2-16-1153-s004.tif]
