# Supplementary material for: Mcl‐1 and Bcl‐xL levels predict responsiveness to dual MEK/Bcl‐2 inhibition in B‐cell malignancies
Source: Mol Oncol. 2021 Dec 18;16(5):1153–70. doi: 10.1002/1878-0261.13153 (PMC8895453; doi:10.1002/1878-0261.13153)
Supplement: Supplementary file 4 — Table S2. Drug combinations. [file MOL2-16-1153-s001.docx]

**Supplementary Table 2.** Drug combinations

| **Drug 1** | **Drug 2** |
| --- | --- |
| Acalabrutinib | Quizartinib |
| Alisertib | Crizotinib |
| AZD6738 | Acalabrutinib |
| Cytarabine | Nutlin 3a |
| Ibrutinib | Chlorambucil |
| Ibrutinib | Fludarabine |
| Ibrutinib | Idelalisib |
| Ibrutinib | Quizartinib |
| Ibrutinib | Selinexor |
| Ibrutinib | SNX-5422 |
| Idelalisib | JQ1 |
| Idelalisib | Quizartinib |
| Idelalisib | Ruxolitinib |
| Idelalisib | Trametinib |
| JQ1 | Palbociclib |
| JQ1 | Ruxolitinib |
| JQ1 | Sorafenib |
| Lenalidomide | Chlorambucil |
| Lenalidomide | Dexamethasone |
| Lenalidomide | Methylprednisolone |
| Palbociclib | Doramapimod |
| Palbociclib | Quizartinib |
| Palbociclib | Ruxolitinib |
| Palbociclib | Sorafenib |
| Palbociclib | Trametinib |
| Ruxolitinib | Cabozantinib |
| Ruxolitinib | Panobinostat |
| Valproic acid | 2-chlorodeoxyadenosine |
| Vandetanib | Vemurafenib |
| Venetoclax | Acalabrutinib |
| Venetoclax | Binimetinib |
| Venetoclax | Cobimetinib |
| Venetoclax | Dasatinib |
| Venetoclax | Duvelisib |
| Venetoclax | Ibrutinib |
| Venetoclax | Idelalisib |
| Venetoclax | Palbociclib |
| Venetoclax | PD0325901 |
| Venetoclax | Pimasertib |
| Venetoclax | Refametinib |
| Venetoclax | Ruxolitinib |
| Venetoclax | Selumetinib |
| Venetoclax | Sorafenib |
| Venetoclax | Trametinib |
| Venetoclax | U0126 |
| Venetoclax | Umbralisib |
