# Supplementary material for: Mcl‐1 and Bcl‐xL levels predict responsiveness to dual MEK/Bcl‐2 inhibition in B‐cell malignancies
Source: Mol Oncol. 2021 Dec 18;16(5):1153–70. doi: 10.1002/1878-0261.13153 (PMC8895453; doi:10.1002/1878-0261.13153)
Supplement: Supplementary file 5 [file MOL2-16-1153-s005.docx]

**SUPPLEMENTARY FIGURE LEGENDS**

**Supplementary Figure 1. Drug sensitivity in OSU-CLL**

a) Freshly thawed OSU-CLL cells were seeded out in 384-well plates pre-printed with a custom-made drug library of 71 single drugs. Cell viability was measured after 72h by CellTiter-Glo. The graph shows average drug sensitivity score (DSS) with standard deviation for n = 2 experiments.

b) Freshly thawed OSU-CLL cells were seeded out in 384-well plates pre-printed with a custom-made drug library of 39 drug combinations. Cell viability was measured after 72h by CellTiter-Glo. The graph shows average drug sensitivity score (DSS) with standard deviation for n = 2 experiments.

**Supplementary Figure 2. Sensitivity to MEK/Bcl-2/Mcl-1/Bcl-xL inhibition in MCL and CLL**

a) Drug sensitivity to the indicated treatments was assessed on 7 MCL cell lines after 72h exposure using CellTiter-Glo. The graph shows relative cell viability. Error bars indicate SEM.

b) Drug sensitivity to the indicated treatments was assessed on 7 MCL cell lines after 72h exposure using CellTiter-Glo. The graph shows relative cell viability. Error bars indicate SEM.

c) Drug sensitivity to the indicated treatments was assessed on 4 CLL cell lines after 72h exposure using CellTiter-Glo. The graph shows relative cell viability. Error bars indicate SEM.
